# Third-Generation Thermodynamic Descriptions for Ta-Cr and Ta-V Binary Systems

**DOI:** 10.3390/ma15062074

**Published:** 2022-03-11

**Authors:** Enkuan Zhang, Xinpei Xu, Yun Chen, Ying Tang

**Affiliations:** 1 School of Materials Science and Engineering, Hebei University of Technology, Tianjin 300130, China; ek_zhang@163.com (E.Z.); xu1020774690@163.com (X.X.); 2 Department of Mechanical and Electrical Engineering, Xiamen University, Xiamen 361005, China; yun.chen@xmu.edu.cn

**Keywords:** third-generation thermodynamic description, Ta-Cr system, Ta-V system, CALPHAD, Laves phase

## Abstract

The third-generation thermodynamic descriptions for Ta-Cr and Ta-V binary systems were performed to construct the reliable thermodynamic database for refractory high-entropy alloys (RHEAs) containing Laves phase. The third-generation Gibbs energy expressions of pure Cr and V in both solid and liquid phases were established, from which the thermodynamic properties and thermal vacancy can be well described. The thermodynamic descriptions of Ta-Cr and Ta-V over the whole composition and temperature regions were carried out based on the reviewed phase equilibria and thermodynamic data with the CALPHAD (CALculation of PHAse Diagrams) approach. Specifically, the thermodynamic parameters of C14 and C15 Laves phases were evaluated by combining the theoretically computed and experimentally measured thermodynamic properties as well as the semiempirical relations. The calculated phase diagrams and thermodynamic properties in Ta-Cr and Ta-V systems according to the present thermodynamic parameters had a nice agreement with the experimental data even down to 0 K, indicating the reliability of the present modeling.

## 1. Introduction

Refractory high-entropy alloys (RHEAs) demonstrate excellent mechanical properties as well as good corrosion resistance and oxidation resistance up to 1600 °C, making them considerable candidates for high-temperature applications [1,2,3,4]. Generally, RHEAs contain refractory elements and form a single solid solution phase with a body-centered cubic (BCC, A2) structure. The addition of the elements, such as Cr and V, which have strong interactions with other refractory elements, can contribute to the formation of Laves phases in RHEAs [5,6,7]. With a minor amount of precipitated Laves phase in A2 solution, the strength of RHEAs can be significantly improved [6,7]. To identify the precipitation of Laves phase in RHEAs, varieties of empirical criteria have been proposed [8,9,10]. With the rapid development of a computational approach, the process of identifying the formation of Laves phase in RHEAs can be sped up to meet unique requirements. Of varied computational methods, the CALPHAD (CALculation of PHAse Diagrams) approach, which can calculate the phase equilibrium in multicomponent alloys, is one of the most powerful methods for Laves phases containing RHEA design and selection [11,12,13]. An accurate thermodynamic database is the prerequisite in CALPHAD-type calculations.

To develop an accurate thermodynamic database over the whole temperature region with physical sense, the concept of a third-generation thermodynamic database has been proposed since 1995 [14]. Under this framework, several research groups [14,15,16,17,18,19] have made efforts to establish new thermodynamic models for pure elements by using the Debye or Einstein models. Recently, a kind of third-generation thermodynamic model [13], which enables one to describe the thermodynamic properties of pure elements down to 0 K as well as thermal vacancy concentration near the melting point, has been proposed by combining the Segmented Regression (SR) model [15], two-state model [20] and thermal vacancy description [18]. By applying such a model, a third-generation thermodynamic database for MoNbTaW RHEAs was developed [13]. To further develop the third-generation thermodynamic databases for Laves containing RHEAs, reliable thermodynamic descriptions for Laves phase are essential. Currently, the reports on third-generation thermodynamic descriptions of Laves phase are rather limited in the literature. Jiang et al. [21,22] established the third-generation Gibbs energy of Laves phase in the Ta-Cr and Cr-Nb systems by considering the experimental heat capacity data with the SR model. Contrastingly, such a strategy was not valid for those systems when the heat capacity data of Laves phase were absent. Thus, a universal strategy to obtain thermodynamic description for Laves phase is needed. 

In this work, the Ta-Cr and Ta-V systems that contain the hexagonal C14 (MgZn_2_-type) and cubic C15 (MgCu_2_-type) Laves phases are selected as the target. Following our previous work [13], the third-generation Gibbs energy expressions of pure Cr and V will be established first. Then, the experimental phase equilibrium and thermodynamic properties in Ta-Cr and Ta-V systems will be reviewed and their discrepancies will be clarified. After that, a universal strategy to obtain reliable thermodynamic parameters in Laves phase will be proposed, and the thermodynamic modeling of the Ta-Cr and Ta-V binary systems will be performed via the CALPHAD approach. 

## 2. Thermodynamic Model

There are four stable phases (i.e., liquid, A2, C14, C15) in Ta-Cr and Ta-V binary systems. The liquid and A2 are the solution phases, while C14 and C15 are the Laves phase. Their thermodynamic models are briefly introduced as follows. 

### 2.1. A2 and Liquid Solution Phases

To describe the thermal vacancy contribution in the A2 phase, the substitutional model (*i*, *j*, *Va*), where *Va* denotes thermal vacancy, was used. The molar Gibbs energy of the A2 phase is as follows:

(1) GmA2=xiGiA2,df+xjGjA2,df+yva1−yvaGva+RT(xilnxi+xjlnxj+yva1−yvalnyva)+(1−yva)xixj∑n=0Li,jA2n(xi−xj)n+11−yva(xiyvaLi,vaA2+xjyvaLj,vaA2)

where *R* and *T* are the gas constant and absolute temperature, $x_{i}$ is the mole fraction of element *i*, and $y_{va}$ is the thermal vacancy concentration. $L_{i,j}$ is the interaction parameter between the elements *i* and *j*, and $L_{i,Va}$ is the one between the element *i* and thermal vacancy. $G_{va}$ represents the Gibbs energy of thermal vacancy in the A2 phase. $G_{i}^{A2,\;df}$ in Equation (1) is the molar Gibbs energy of the defect-free element *i*, the expression of which will be developed by using the SR model [15]. For the liquid phase, its molar Gibbs energy expression is similar to that of the A2 phase but excludes the contribution from the thermal vacancy (to make *y_va_* = 0 in Equation (1)). The Gibbs energy of pure element in a liquid state will be established by applying the two-state model [20].

### 2.2. C14 and C15 Laves Phases 

A two-sublattice model (*i*, *j*)_2_ (*i*, *j*) was employed to describe the solid solubility in C14 and C15 phases. Take the C15 phase in Ta-Cr binary, for example; its molar Gibbs energy is given as follows:

(2) GC15=G0Cr:TaC15yCr′yTa″+G0Cr:CrC15yCr′yCr″+G0Ta:TaC15yTa′yTa″+G0Ta:CrC15yTa′yCr″+2RT(yCr′lnyCr′+yTa′lnyTa′)+RT(yCr″lnyCr″+yTa″lnyTa″)+yCr′yCr″yTa″L0Cr:Cr,TaC15+yTa′yCr″yTa″L0Ta:Cr,TaC15+yCr′yTa′yCr″L0Cr,Ta:CrC15+yCr′yTa′yTa″L0Cr,Ta:TaC15

where $y_{i}^{\prime }$ and $y_{i}^{\prime \prime }$ denote the site fractions in the first and second sublattice of element *i*. G0i:jC15 is the end-member Gibbs energy when the first sublattice is occupied by *i* while the second sublattice is occupied by *j*. Its expression is given as

(3) G0i:jC15=A+B⋅T+2⋅GiA2,def+GjA2,def

where *A* and *B* are the coefficients to be optimized. L0Cr:i.jC15 and L0Ta:i.jC15 are the interaction parameters between *i* and *j* in the second sublattice when the first is occupied by Cr or Ta. Similarly, L0i.j:CrC15 and L0i.j:TaC15 represent the interaction parameters between *i* and *j* in the first sublattice when the second sublattice is occupied by Cr or Ta, which are all also needed to be determined during the thermodynamic assessment. 

## 3. Results

### 3.1. The Third-Generation Gibbs Energy Expressions for Pure Cr and V 

The reliable thermodynamic properties of pure Cr and V are the essential inputs to establish their Gibbs energy expressions. Recently, the measured thermodynamic properties including heat capacity (*C_p_*), heat content (*H*_T_–*H*_298_), enthalpy of fusion for pure Cr and V have been collected and reviewed by Obaied et al. [19] and Arblaster [23], respectively. The discrepancies of the measured data from different resources have been clearly clarified by them. Therefore, the reviewed thermodynamic properties are adopted in the present modeling. In addition, the thermal vacancy concentrations in pure Cr and V at their melting temperature were experimentally investigated by means of modulation and drop methods [24,25]. It is generally accepted that the effects of thermal vacancy on thermodynamic properties will become obvious with the temperature above 2/3*T_m_* [18]. Thus, the expressions of defect-free Gibbs energy expressions ($G_{i}^{A2,df}$) in Equation (1) of pure Cr and V in their solid stable states (below *T_m_*) were evaluated by fitting the experimental heat capacity from 0 K to 2/3*T_m_*. As for the expressions above *T_m_*, the strategy making $C_{p}^{}$ and $dC_{p}^{}/dT$ curves continue at *T_m_* was used. Then, the interaction parameter $L_{i,Va}$ was evaluated by considering the thermodynamic properties above 2/3*T_m_* as well as the thermal vacancy concentration at melting point. The Gibbs energy of the A2 phase with thermal vacancy over the whole temperature range for Cr and V can be evaluated by combining the estimated $G_{i}^{A2,df}$ and $L_{i,Va}$.The molar Gibbs energies of pure Cr and V in a liquid state were obtained with a two-state model by considering the experimental entropy, enthalpy and heat capacity at and above *T_m_*. The finally obtained third-generation Gibbs energies for solid and liquid Cr and V are listed in Table 1.

**Figure 1:**
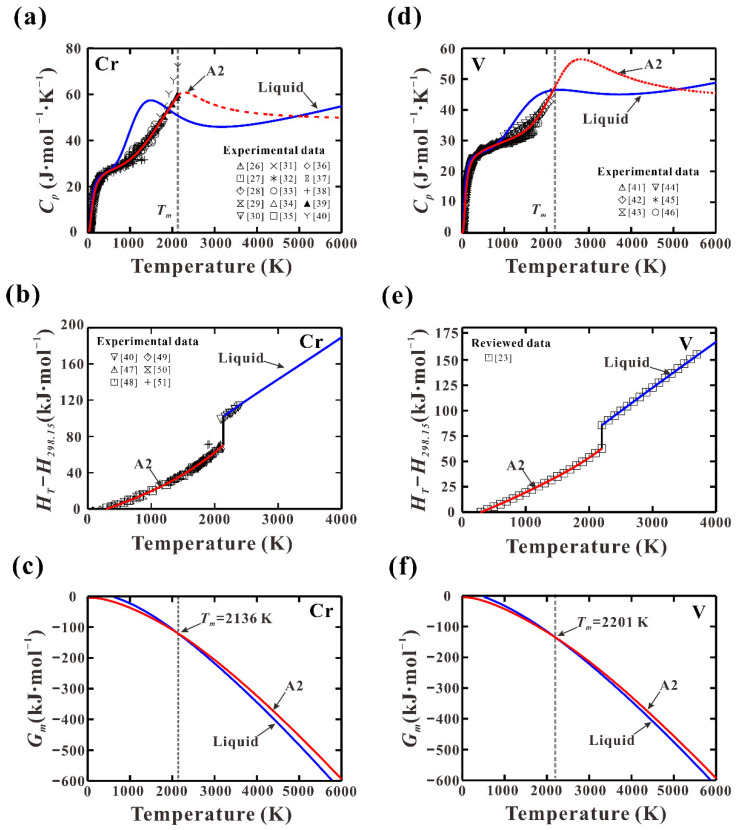
Calculated thermodynamic properties: (**a**,**d**) heat capacity (*C_p_*); (**b**,**e**) heat content (*H*_T_–*H*_298_); (**c**,**f**) molar Gibbs energy of pure Cr and V in comparison with reported data [23,26,27,28,29,30,31,32,33,34,35,36,37,38,39,40,41,42,43,44,45,46,47,48,49,50,51].

Figure 1a,d show the calculated *C_p_* of pure Cr and V in their solid A2 and liquid states. The experimental *C_p_* data [26,27,28,29,30,31,32,33,34,35,36,37,38,39,40,41,42,43,44,45,46] are also appended in the figure for comparison, showing a nice agreement with present calculations. Figure 1b,d show the calculated *H*_T_-*H*_298_ of pure Cr and V from 298 to 4000 K together with the reported data [23,47,48,49,50,51]. An excellent agreement can be observed. The Gibbs energies of solid A2 and liquid phases in pure Cr and V from 0 K to 6000 K were calculated and displayed in Figure 1c,f, revealing the lattice stability over the whole temperature range.

With the present established Gibbs energies, the thermal vacancy can be the equilibrium concentration of thermal vacancy in pure Cr, and V can be predicted by the following relation [18]:

(4) $$y_{va}=\exp(-\frac{G_{i}^{A2,df}+L_{i,va}^{A2}{\left(1-y_{va}\right)}^{2}}{RT})$$

**Figure 2:**
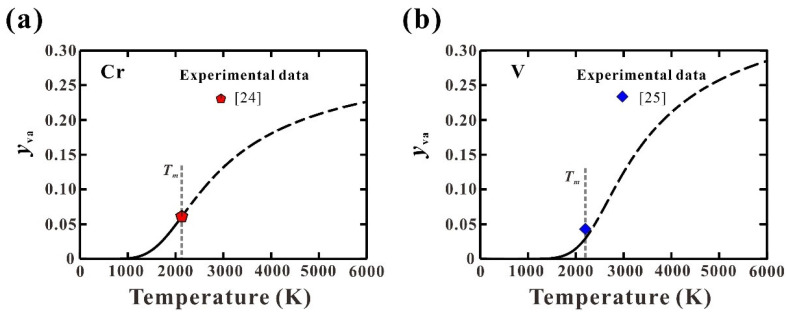
Calculated thermal vacancy concentrations in pure solid (**a**) Cr and (**b**) V along with measured data [24,25] at melting points.

Figure 2 shows the calculated thermal vacancy concentrations in solid Cr and V. As can be seen, the thermal vacancy concentrations increase with the increase in temperatures, and a dramatic increase can be observed near the melting points. Besides, the present predicted thermal vacancy concentrations are in good agreement with those from measurements [24,25] at melting points. The above results indicate the reliability of the established third-generation Gibbs energies for pure Cr and V. 

### 3.2. Thermodynamic Descriptions of Ta-Cr and Ta-V Binary Systems 

Ta-Cr and Ta-V binary systems were thermodynamically assessed by several research groups [52,53,54,55,56]. In these thermodynamic modeling, the Gibbs energy of pure elements were taken from SGTE (Scientific Group Thermodata Europe) data [57], which is also known as the second generation of thermodynamic description. In this description, the Gibbs energy was expressed as empirical temperature polynomials and valid only down to 298 K. In this section, the Ta-Cr and Ta-V binary systems were thermodynamically re-assessed by utilizing the above established third-generation Gibbs energy expressions for pure Cr and V as well as those for Ta from our previous work [13]. In addition, an effective strategy to obtain Gibbs energy for Laves phase was proposed and applied to C14 and C15 Laves phases in Ta-Cr and Ta-V systems.

Since the experimental phase equilibrium and thermodynamic properties in the Ta-Cr binary system have been recently reviewed by Jiang et al. [22], their reviewed data were adopted in the present modeling. As for the Ta-V binary system, most of the reported data have been reviewed by Danon and Servant [55] (2004). Thus, only the latest experimental data in the Ta-V binary system available in the literature will be briefly summarized and their discrepancies will be clarified. The greatest controversy in the Ta-V binary system is focused on the phase transition between C14 and C15 Laves phases. By reviewing the existing experimental data, Danon and Servant [55] concluded that C14 existed at a high temperature, while C15 existed at a low temperature, and there was a narrow (C15 + C14) two-phase region from 1400 to 1548 K. In addition, the eutectoid (C14 → C15+A2) and peritectoid (C14 + A2 → C15) reactions can be found in the V-rich and Ta-rich sides. After that, Pavlů et al. [56] pointed out that the C14 phase was more stable than C15 at 0 K based on their ab initio calculations. Thus, in their thermodynamic modeling, the C14 existed in two temperature regions, that is, from 0 to 626 K and from 1409 to 1703 K. Later, the measurements from Khan et al. [58] indicated that C14 was not found in four Ta-V alloys, which were annealed at 1473 K for 15 days. However, the recent phase equilibrium investigations in ternary Ta-V-Ni [59] and Ta-V-Co [60] systems displayed that the C14 phase existed in the Ta-V side at 1473 and 1573 K but was not found at 1173, 1273 and 1373 K after long-time annealing. Since no further experimental data confirm the existence of C14 below 626 K, as proposed by Pavlů et al. [56], the phase relationship between C14 and C15 recommended by Danon and Servant [55] was adopted in the present modeling.

During the thermodynamic modeling, the interaction parameters of the liquid and A2 phases were evaluated by considering the liquidus, solidus temperatures and invariant reactions. To obtain accurate thermodynamic descriptions for C14 and C15 Laves phases, the values of end-members in Equation (2) should be fixed initially. In this work, the values of G0Cr:Ta and G0V:Ta were evaluated by considering the experimental thermodynamic properties referenced to Cr2Ta and V2Ta alloys, which generally locate in the stable C14 or C15 phase region. The endmembers G0Cr:Cr,G0Ta:Ta and G0V:V are the Gibbs energies of pure elements with MgZn2-type (C14) and MgCu2-type (C15) crystal structures, which are the metastable ones, and their values cannot be directly obtained from the experimental investigation. Sluiter [61] computed the formation energies of a great deal of pure elements at 0 K in a variety of structures, including C14 and C15 by using electronic density functional theory (DFT). These theoretically predicted values can be served as start values for G0Cr:Cr,G0Ta:Ta and G0V:V. To satisfy the Wagner–Schottky defects, there should be a constraint among the end-members when applying the two-sublattice model, that is, G0i:i+G0j:j=G0i:j+G0j:i [62]. In the present work, the values of G0Ta:Cr and G0Ta:V are obtained by applying the above constraint. The interaction parameters in C14 and C15 Laves phase were adjusted to reproduce the invariant reactions. The thermodynamic parameters of Ta-Cr and Ta-V binary systems are summarized in Table 2.

**Figure 3:**
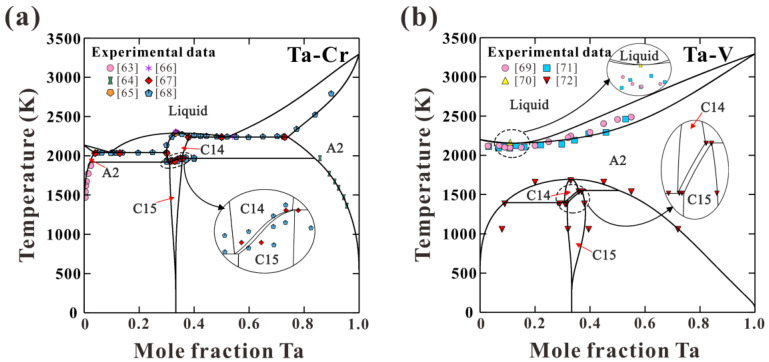
Calculated phase diagrams for (**a**) Ta-Cr and (**b**) Ta-V binary systems along with experimental data [63,64,65,66,67,68,69,70,71,72].

**Figure 4:**
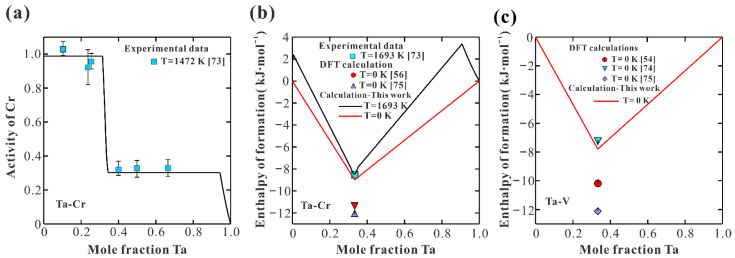
Calculated thermodynamic properties in Ta−Cr and Ta-V systems together with reported data [54,56,73,74,75]: (**a**) activity of Cr in Ta-Cr system at 1472 K; (**b**) enthalpy of formation in Ta-Cr system at 0 and 1693 K; (**c**) enthalpy of formation in Ta-V system at 0 K.

Figure 3 shows the calculated phase diagrams of Ta-Cr and Ta-V binary systems according to the present thermodynamic descriptions. It shows that most of the experimental data [63,64,65,66,67,68,69,70,71,72] can be well reproduced. There are two invariant reactions between C14 and C15 Laves phases in Ta-Cr system, including a eutectoid reaction C14 → A2(Cr) + C15 and a peritectoid reaction (C14 + A2(Ta) → C15), as shown in the enlarged area in Figure 3a. A similar phase transition between C14 and C15 Laves phases in the Ta-V system can be observed. There is an azeotropic melting point in the Ta-V binary system, as shown in Figure 3b. The composition and temperature of this azeotropic melting point were calculated to be 12.4 at.% Ta and 2156 K, which were close to the measured ones [68,69,70]. Table 3 summarizes the calculated compositions and temperatures of the invariant reactions in Ta-Cr and Ta-V systems together with those from measurements [67,68,69,70,71,72] and previous calculations [53,54,55,56]. The present calculations agree reasonably with most of the measured data. Besides, the present results are quite close to those from previous assessments [53,54,55,56]. Figure 4 (a) presents the calculated activity of Cr over the whole composition range in the Ta-Cr system at 1472 K compared with the experimental data [73]. As can be seen, the calculations are within the uncertainty range of the experimental data. Figure 4b shows the calculated enthalpy of formation in the Ta-Cr system at 0 and1693 K, and the measured data [73] and DFT calculations [54,56,73,74,75] are also appended for a comparison. The calculated enthalpy formation of the C15 phase at 1693 K has a nice agreement with that from measurement [73]. In addition, the calculated enthalpy of formation at 0 K is more negative than that at 1693 K. Figure 4c gives the calculated enthalpy formation in the Ta-V system at 0 K. Since the theoretically computed formation enthalpy in the C15 phase from different sources is rather scattered, the present calculated result is reasonable.

**Figure 5:**
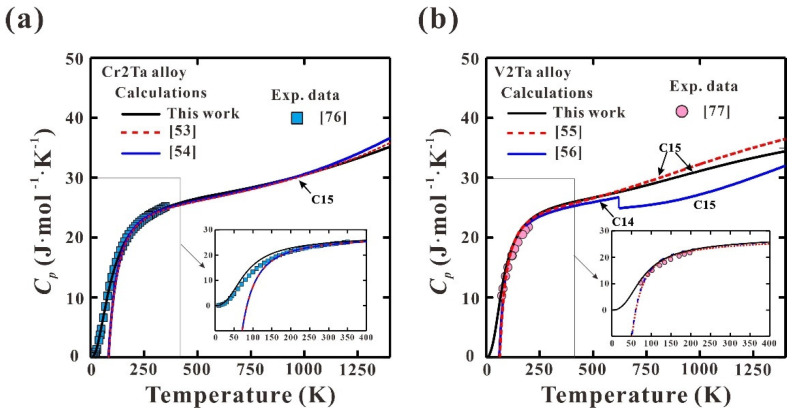
Calculated *C_p_* for (**a**) Cr2Ta and (**b**) V2Ta alloys from 0 to 1400 K along with the reported data [76,77].

Figure 5 shows the calculated *C_p_* of Cr2Ta and V2Ta alloys from 0 to 1400 K. Based on the present modeling, Cr2Ta and V2Ta alloys locate in the C15 single phase region below 1948.6 and 1446.7 K. Although the experimental *C_p_* data were not considered during the modeling, the present calculations show a nice agreement with the reported data [76,77]. The calculations from previous thermodynamic assessments [52,53,54,55,56] are also presented in Figure 5 for a comparison. As shown in Figure 5a, the calculated *C_p_* of Cr2Ta from [53,54] are quite close to the present calculated ones at high temperatures, while obvious deviations can be observed at low temperatures (below 298 K). The present calculations show better agreement with the measured ones. In addition, the values of *C_p_* for Cr2Ta and V2Ta alloys, according to the previous assessments [53,54,55,56], decrease rapidly as the temperature decreases and become negative at 85 and 62 K, which is physically inaccurate. It also implies that the present thermodynamic description of C15 Laves phase in Ta-Cr and Ta-V binary systems is reliable. It is highly believed that the present strategy to establish the thermodynamic descriptions of Laves phase is reliable, which can be applied to develop the third-generation thermodynamic database for Laves phases containing RHEAs.

## 4. Conclusions

- The third-generation Gibbs energy expressions of pure Cr and V in both the liquid and A2 phases were established. By applying these expressions, the thermodynamic properties down to 0 K and thermal vacancy near the melting point can be well described. Besides, the lattice stability of Cr and V over the whole temperature range can be guaranteed.
- Based on the third-generation Gibbs energy expressions of pure elements, the Ta-Cr and Ta-V binary systems were thermodynamically assessed by considering the reviewed phase equilibria and thermodynamic data with the CALPHAD approach. A strategy to estimate the Gibbs energy of Laves phase was proposed by combining the theoretically computed and experimentally measured thermodynamic properties as well as semiempirical relation. Such a method was applied to C14 and C15 Laves phases in Ta-Cr and Ta-V binary systems. The calculated phase diagrams and thermodynamic properties showed nice agreement with the measured ones. Significant improvements can be observed at low temperatures compared with those from the second-generation thermodynamic descriptions, indicating the high reliability of the present thermodynamic descriptions.

## Figures and Tables

**Table 1:** The third-generation Gibbs energy expressions for pure Cr and V in solid A2 and liquid phases.

Element	Phases	Gibbs Energy (J/mol)
Cr	A2	$G_{Cr}^{A2,\;df}=\left\{ \begin{array}{ll} -13245.18-2.89\times 10^{-3}\cdot T^{2}+16.21T\ln(e^{432.5532/T}-1)+8.71T\ln(e^{251.1734/T}-1) & \;\;\;0<T<531.9\\ -13745.71+14.84T+1.26\times 10^{-3}T^{2}-1.12\times 10^{-6}T^{3}-2.52T\ln\left(T\right)+16.21T\ln(e^{432.5532/T}-1)+8.71T\ln(e^{251.1734/T}-1) & 531.9<T<2136\\ -38598.14+188.46\cdot T-23.90T\ln\left(T\right)-5.42\times 10^{19}T^{-5}+9.03\times 10^{38}T^{-11}+16.21T\ln(e^{432.5532/T}-1)+8.71T\ln(e^{251.1734/T}-1) & \;\;\;T>2136 \end{array}\right.$ $G_{VA}^{A2}=+30T$ $;\;L_{Cr,VA}^{A2}=+72220-41.50T$
	Liquid	$G_{Cr}^{liq}=14164.90+24.942\cdot T\cdot \ln(1-e^{-297.2382/T})-2.23\times 10^{-3}T^{2}-8.314\cdot T\cdot \ln(1+e^{(45995.32-14.8855\cdot T-1.16\times 10^{-1}\cdot \ln(T))/8.314T})$
V	A2	$G_{V}^{A2,\;df}=\left\{ \begin{array}{ll} -11655.70-2.50\times 10^{-3}T^{2}+23.58T\ln(e^{289.5879/T}-1)+1.29T\ln(e^{90.48463/T}-1) & 0<T<2201\\ -42621.43+144.41T-17.69T\ln\left(T\right)+4.38\times 10^{19}T^{-5}-4.76\times 10^{38}T^{-11}+23.58T\ln(e^{289.5879/T}-1)+1.29T\ln(e^{90.48463/T}-1) & \;\;\;\;\;\;\;T>2201 \end{array}\right.$ $G_{VA}^{A2}=+30T$ $;\;L_{V,VA}^{A2}=+130500-60.01T$
	Liquid	$G_{V}^{liq}=11355.04+24.942\cdot T\cdot \ln(1-e^{-205.2036/T})-1.77\times 10^{-3}T^{2}-8.314\cdot T\cdot \ln(1+e^{(53359.83-10.4607\cdot T-5.66\times 10^{-1}\cdot \ln(T))/8.314T})$

**Table 2:** Summary of the thermodynamic parameters of Ta-Cr and Ta-V binary systems.

Systems	Phases	Thermodynamic Parameters (J/mol)
Ta-Cr	Liquid	L0Cr,TaLiquid=+5647.3−4.576T;L1Cr,TaLiquid=+10555.4−1.121T;L2Cr,TaLiquid=−21769.6+3.718T
	A2	L0Cr,TaA2=+84411.6−30.246T;L1Cr,TaA2=+50274.2−21.825T;L2Cr,TaA2=−4321.6
	C14	G0Cr:CrC14=+83400.0+3GCrA2,dfG0Ta:TaC14=+29100.0+3GTaA2,dfG0Cr:TaC14=−19550.7−5.770T+2GCrA2,df+GTaA2,dfG0Ta:CrC14=+132050.7+5.536T+GCrA2,df+2GTaA2,dfL0Cr,Ta:*C14=+219623.9−74.407T;L0*:Cr,TaC14=+95266.4−77.887T
	C15	G0Ta:TaC15=+33600.0+3GTaA2,dfG0Cr:CrC15=+79200.0+3GCrA2,dfG0Cr:TaC15=−26555.2−2.170T+2GCrA2,df+GTaA2,dfG0Ta:CrC15=+139755.2+1.813T+GCrA2,df+2GTaA2,dfL0Cr,Ta:*C15=+51828.2+8.181T;L0*:Cr,TaC15=−61398.1+7.891T
Ta-V	Liquid	L0V:TaLiquid=−5751.2; L1V:TaLiquid=−3341.9
	A2	L0V:TaA2=+3731.5; L1V:TaA2=−10998.9
	C14	G0Ta:TaC14=+29100.0+3GTaA2,dfG0V:VC14=+28800.0+3GVA2,dfG0V:TaC14=−18925.2+2GVA2,df+GTaA2,dfG0Ta:VC14=+76825.2+GVA2,df+2GTaA2,dfL0V,Ta:*C14=−11321.2;L0*:V,TaC14=−6751.6
	C15	G0Ta:TaC15=+33600.0+3GTaA2,dfG0V:VC15=+33000.0+3GVA2,dfG0V:TaC15=−23389.9+3.004T+2GVA2,df+GTaA2,dfG0Ta:VC15=+89989.9−3.004T+GVA2,df+2GTaA2,dfL0V,Ta:*C15=−349.549+1.025T;L0*:V,TaC15=−7515.567

**Table 3:** Calculated temperatures and compositions of invariant reactions in Ta-Cr and Ta-V binary systems in comparison with the measured ones [67,68,69,70,71,72] and previous calculated ones [53,54,55,56].

Systems	Reactions	Temperature (K)	Composition (at. %Ta)			Ref.
Ta-Cr	Liquid → C14	2293.0 ± 20	--	33.30	--	[67] (Exp.)
		2309.7	--	34.90	--	[53] (Cal.)
		2304	--	34.22	--	[54] (Cal.)
		2290.0	--	34.87	--	This work (Cal.)
	Liquid → A2(Cr) + C14	2033.0 ± 20	~13.00	~4.00	~30.00	[67] (Exp.)
		2040.0 ± 10	~10.50	~3.50	~30.00	[68] (Exp.)
		2044.7	11.53	4.53	30.03	[53] (Cal.)
		2065.1	9.88	3.89	30.65	[54] (Cal.)
		2041.9	9.59	3.68	29.94	This work (Cal.)
	Liquid → A2(Ta) + C14	2238.0 ± 20	~50.0	~73.00	~38.00	[67] (Exp.)
		2223.4	49.41	72.75	37.90	[53] (Cal.)
		2239.2	49.96	73.32	37.70	[54] (Cal.)
		2239.0	52.12	74.13	38.21	This work (Cal.)
	C14 → A2(Cr) + C15	1933.0	~31.40	--	~33.00	[67] (Exp.)
		1917.2	30.96	2.81	32.23	[53] (Cal.)
		1903.3	30.97	1.87	31.11	[54] (Cal.)
		1921.0	30.83	2.17	31.15	This work (Cal.)
	C14 + A2(Ta) → C15	1968.0	~35.00	--	~36.00	[67] (Exp.)
		1982.9	35.17	80.58	35.55	[53] (Cal.)
		1991.2	37.29	84.72	37.39	[54] (Cal.)
		1969.0	35.52	83.86	35.72	This work (Cal.)
Ta-V	Liquid → A2	2153.0	--	11.00	--	[70] (Exp.)
		2098.0	--	15.00	--	[69] (Exp.)
		--	--	12.00	--	[68] (Exp.)
		2099	--	12.89	--	[55] (Cal.)
		2099	--	12.89	--	[56] (Cal.)
		2156.0	--	12.40	--	This work (Cal.)
	A2 → C14	1693.0	--	~33.00	--	[71] (Exp.)
		1702.2	--	32.70	--	[55] (Cal.)
		1703.3	--	32.62	--	[56] (Cal.)
		1693.0	--	~33.00	--	[72] (Exp.)
		1695.1	--	33.22	--	This work (Cal.)
	C14 + A2(Ta) → C15	1553.0	36.00	37.00	55.00	[72] (Exp.)
		1550.2	35.97	37.36	50.27	[55] (Cal.)
		1556.1	36.14	37.78	57.21	[56] (Cal.)
		1552.5	35.87	37.02	50.46	This work (Cal.)
	C14 → A2(V) + C15	1398.0	29.00	9.00	31.50	[72] (Exp.)
		1403.0	31.02	6.48	31.29	[55] (Cal.)
		1409.5	30.69	3.41	31.64	[56] (Cal.)
		1396.0	30.70	8.90	31.85	This work (Cal.)

## Data Availability

The data presented in this study are available on request from the corresponding author.
